# Genome-wide pleiotropy analysis of neuropathological traits related to Alzheimer’s disease

**DOI:** 10.1186/s13195-018-0349-z

**Published:** 2018-02-20

**Authors:** Jaeyoon Chung, Xiaoling Zhang, Mariet Allen, Xue Wang, Yiyi Ma, Gary Beecham, Thomas J. Montine, Steven G. Younkin, Dennis W. Dickson, Todd E. Golde, Nathan D. Price, Nilüfer Ertekin-Taner, Kathryn L. Lunetta, Jesse Mez, Alzheimer’s Disease Genetics Consortium, Richard Mayeux, Jonathan L. Haines, Margaret A. Pericak-Vance, Gerard Schellenberg, Gyungah R. Jun, Lindsay A. Farrer

**Affiliations:** 1 Bioinformatics Graduate Program, Boston University, Boston, MA USA; 2 Department of Medicine (Biomedical Genetics), Boston University School of Medicine, Boston, MA USA; 3 Department of Neuroscience, Mayo Clinic, Jacksonville, FL USA; 4 Department of Health Sciences Research, Mayo Clinic, Jacksonville, FL USA; 5 Hussman Institute for Human Genomics, University of Miami Miller School of Medicine, Miami, FL USA; 6 Department of Pathology, University of Washington, Seattle, WA USA; 7 Center for Translational Research in Neurodegenerative Disease, McKnight Brain Institute, University of Florida, Gainesville, FL USA; 8 Institute for Systems Biology, University of Washington, Seattle, WA USA; 9 Department of Neurology, Mayo Clinic, Jacksonville, FL USA; 10 Department of Biostatistics, Boston University School of Public Health, Boston, MA USA; 11 Department of Neurology, Boston University School of Medicine, Boston, MA USA; 12 Department of Neurology and Sergievsky Center, Columbia University, New York, NY USA; 13 Department of Epidemiology and Biostatistics, Case Western Reserve University, Cleveland, OH USA; 14 Department of Pathology and Laboratory Medicine, University of Pennsylvania, Philadelphia, PA USA; 15 Neurogenetics and Integrated Genomics, Andover Innovative Medicines Institute, Eisai Inc., Andover, MA USA; 16 Department of Ophthalmology, Boston University School of Medicine, Boston, MA USA; 17 Department of Epidemiology, Boston University School of Public Health, Boston, MA USA

**Keywords:** Alzheimer’s disease, Neuropathological traits, Genome-wide association study, Pleiotropy analysis, *HDAC9*, *ECRG4*

## Abstract

**Background:**

Simultaneous consideration of two neuropathological traits related to Alzheimer’s disease (AD) has not been attempted in a genome-wide association study.

**Methods:**

We conducted genome-wide pleiotropy analyses using association summary statistics from the *Beecham et al*. study (PLoS Genet 10:e1004606, 2014) for AD-related neuropathological traits, including neuritic plaque (NP), neurofibrillary tangle (NFT), and cerebral amyloid angiopathy (CAA). Significant findings were further examined by expression quantitative trait locus and differentially expressed gene analyses in AD vs. control brains using gene expression data.

**Results:**

Genome-wide significant pleiotropic associations were observed for the joint model of NP and NFT (NP + NFT) with the single-nucleotide polymorphism (SNP) rs34487851 upstream of *C2orf40* (alias *ECRG4*, *P* = 2.4 × 10^−8^) and for the joint model of NFT and CAA (NFT + CAA) with the *HDAC9* SNP rs79524815 (*P* = 1.1 × 10^−8^). Gene-based testing revealed study-wide significant associations (*P* ≤ 2.0 × 10^−6^) for the NFT + CAA outcome with adjacent genes *TRAPPC12*, *TRAPPC12-AS1*, and *ADI1*. Risk alleles of proxy SNPs for rs79524815 were associated with significantly lower expression of *HDAC9* in the brain (*P* = 3.0 × 10^−3^), and *HDAC9* was significantly downregulated in subjects with AD compared with control subjects in the prefrontal (*P* = 7.9 × 10^−3^) and visual (*P* = 5.6 × 10^−4^) cortices.

**Conclusions:**

Our findings suggest that pleiotropy analysis is a useful approach to identifying novel genetic associations with complex diseases and their endophenotypes. Functional studies are needed to determine whether *ECRG4* or *HDAC9* is plausible as a therapeutic target.

**Electronic supplementary material:**

The online version of this article (10.1186/s13195-018-0349-z) contains supplementary material, which is available to authorized users.

## Background

Alzheimer’s disease (AD) is the most common type of dementia in persons aged 65 years and older [1, 2]. Pathologically, it is characterized primarily by the appearance of both neuritic plaques (NPs) containing oligomers of β-amyloid and neurofibrillary tangles (NFTs), accompanied by a progressive loss of neurons in the brain [3, 4]. Also, cerebral amyloid angiopathy (CAA), which is caused by aggregates of β-amyloid in walls of blood vessels in the brain, is found in as many as 90% of autopsy-confirmed AD cases [5]. Previously, *Beecham et al.* identified multiple significant gene associations in a genome-wide association study (GWAS) for several AD-related neuropathological traits, including NP, NFT, and CAA measured in brains from subjects with pathologically confirmed AD cases and from control subjects with no evidence of neurological disease [6]. We hypothesized that additional novel associations could be identified in models allowing a genetic variant to influence more than one trait (i.e., pleiotropy). In this study, we performed genome-wide pleiotropy analyses of joint models of NP, NFT, and CAA using summary data from the previous study [6].

## Methods

### Study population, neuropathological trait selection, and data processing

We obtained summary statistics from univariate GWAS of NP, NFT, and CAA [6]. These results were derived from meta-analyses of 12 studies including 3598 subjects (3135 AD cases, 463 controls) of European ancestry. Neuropathological data for the entire sample were reviewed and harmonized by one neuropathologist for consistency across studies [6]. Although *Beecham et al.* [6] also evaluated Lewy body disease, hippocampal sclerosis, and vascular brain disease, we limited our present analyses to neuropathological outcomes most directly linked to AD and moderately correlated with each other (i.e., NP, NFT, and CAA). Uncorrelated traits are unlikely to show significant pleiotropic associations, and results from pleiotropy analysis will be similar to those from univariate models (i.e., single phenotype) if the traits are highly correlated. Details of subject recruitment, genotyping, genotype imputation, quality control procedures, population substructure analysis, and statistical methods for association analyses of individual traits were reported previously [6, 7]. Sample demography of the 3598 subjects with autopsied brains and genotypes (3135 cases and 463 controls) is described in Additional file 1: Table S1.

### Univariate genome-wide association analyses

Results from the association tests by *Beecham et al*. [6] in each dataset for each neuropathologic trait with genotypes imputed using the 1000 Genomes Project reference panel (GRCh37 at December 2010) for a genome-wide set of single-nucleotide polymorphisms (SNPs) were obtained using ordinal logistic regression models including the first three principal components of ancestry as covariates to account for population substructure [6]. NP and NFT measures were analyzed in well-established ordinal rankings (NPs: none, sparse, moderate, and frequent by Consortium to Establish a Registry for Alzheimer’s Disease [“CERAD”] scoring [8]; NFT: none, transentorhinal, limbic, and isocortical by Braak and Braak staging [9]), and CAA was analyzed as a binary trait (present or absent). Full details of these analyses are reported elsewhere [6]. We used GWAS meta-analysis summary statistics (β and SE) of the three neuropathologic traits for 6.5 million imputed SNPs after omitting SNPs from studies if the minor allele frequency was ≤ 1%, imputation quality (*R*^2^) was ≤ 0.4, or dosage variance was ≤ 0.02.

### Genome-wide pleiotropy analyses

We conducted a genome-wide pleiotropy analysis for each pair of the three neuropathological traits using the O’Brien method [10, 11], which is implemented in an R library (“CUMP”) [12]. This method combines univariate test statistics (Z-scores from β and SE values) of all SNPs from separate GWASs for individual phenotypes to compute a test statistic that follows a multivariate normal distribution. The covariance matrix of the distribution was approximated by the sample covariance matrix of the test statistics of all SNPs. Under the null hypothesis, an SNP is not associated with any of the phenotypes. The alternative hypothesis is that an SNP is associated with at least one of the phenotypes. We defined a SNP as having a pleiotropic effect on two phenotypes when the *P* value for the O’Brien test statistic from the joint model of association of two phenotypes (*P*_joint_) with the SNP is at least one order of magnitude more significant than the *P* values (*P*_univariate_) for both phenotypes and the univariate *P* values are at least nominally significant (*P*_univariate_ < 0.05). As a supplementary analysis, we also conducted a trivariate pleiotropy genome-wide analysis for the three neuropathological traits. The genome-wide significance (GWS) threshold for these analyses was set at *P* < 5.0 × 10^−8^.

### Gene-based association

We performed genome-wide gene-based tests for each joint model using results from individual SNP tests. SNPs within 30 kb of the transcription start and end sites were included in each gene-based test. These analyses were carried out using the versatile gene-based test (“VEGAS”) method [13], which computes an empirical *P* value through Monte Carlo simulations based on linkage disequilibrium patterns of the European ancestry population in the 1000 Genomes Project (GRCh37 released March 2012). The GWS level for the gene-based tests was set at 2.7 × 10^−6^, which was calculated as the nominal significance level 0.05 divided by the total number of genes tested (*n* = 18,500).

### Expression quantitative trait locus analysis

The association of SNP genotypes with gene-level expression (i.e., expression quantitative trait loci [eQTLs]) was evaluated using version 6 of the GTEx Portal database (http://www.gtexportal.org/; [14]) and data from the Mayo Clinic brain expression GWAS (eGWAS) (https://www.synapse.org/#!Synapse:syn3157249 or http://alois.med.upenn.edu/niagads; [15]). The GTEx Portal provides eQTL association summary statistics (β and *P* values) across 43 different tissues from 175 subjects. The Mayo Clinic brain eGWAS data were generated from the cerebellum (CER; 197 AD and 177 non-AD control subjects) and temporal cortex (TCX; 202 AD and 197 non-AD control subjects) regions. Gene expression measures for 24,526 probes were generated with the Illumina Whole Genome DASL array (Illumina, San Diego, CA, USA). SNP genotype data for the Mayo Clinic eGWAS were obtained from the Mayo Clinic late-onset AD GWAS [16]. AD cases were diagnosed as definite AD according to National Institute of Neurological and Communicative Disorders and Stroke/Alzheimer’s Disease and Related Disorders Association criteria, whereas non-AD controls had other neuropathologies. For each brain region, association of gene expression and imputed SNP genotype (GRCh36) was evaluated using linear regression, including covariates for AD status, apolipoprotein E (*APOE)* ε4 dosage (0, 1, or 2), age at death, sex, plate, RNA integrity number (RIN), and adjusted RIN (RIN − RINmean^2^). Analyses were also conducted for AD cases and controls separately.

### Differential gene expression analysis

Differential gene expression (DGE) analysis was performed using publicly available brain whole-transcriptome RNA-sequencing (RNA-Seq) data [17] and microarray data (Gene Expression Omnibus accession number [GEO:GSE44772] [18]). The RNA-Seq data include DGE summary statistics for the CER and TCX derived from 86 patients with AD and 80 control subjects (https://www.synapse.org). Following a quality control step, 80 AD and 76 control brains were analyzed. All subjects underwent RNA-Seq using the Illumina HiSeq 2000 sequencing system (101 bp, paired-end sequencing) at the Mayo Clinic Genomic Core Facility. All AD and some of the control brains were from the Mayo Clinic Brain Bank, whereas other control brains were from the Banner Sun Health Research Institute (Sun City, AZ, USA). Following quality control, raw read counts normalized according to conditional quantile normalization (CQN) employing the Bioconductor package were used in the analyses. For DGE comparing AD with controls, multivariable linear regression analyses were conducted in R, using CQN normalized gene expression measures and including age at death, sex, RIN, brain tissue source, and flow cell as biological and technical covariates. To account for any CNS cell-population changes that occur as a consequence of disease pathology, we also included cell-specific gene levels as covariates, using the expression levels for the five central nervous system (CNS)-specific genes as follows: *ENO2* for neurons [ENCODE:ENSG00000111674], *GFAP* for astrocytes [ENCODE:ENSG00000131095], *CD68* for microglia [ENCODE:ENSG00000129226], *OLIG2* for oligodendrocytes [ENCODE:ENSG00000205927], and *CD34* for endothelial cells [ENCODE:ENSG00000174059].

The microarray gene expression data were generated from autopsied brains collected from dorsolateral prefrontal cortex (DLPFC), visual cortex (VCX), and CER regions of 129 AD patients and 101 control subjects. Samples were profiled on a custom-made Agilent 44K array (Agilent Technologies, Santa Clara, CA, USA) containing 40,638 human genes. Gene expression data were normalized using Rosetta Resolver gene expression analysis software as previously described [18]. The association between expression of each gene (outcome) and AD status (predictor) was tested using linear regression adjusting for RIN, postmortem interval, batch, preservation method, pH in tissues, age, sex, and the five cell-type markers.

## Results

NP, NFT, and CAA were moderately correlated (NP-NFT, *r* = 0.68; NP-CAA, *r* = 0.56; NFT-CAA, *r* = 0.40; *P* < 2.2 × 10^−16^ for each pair of traits), indicating a potential for discovery of novel associations in pleiotropy analysis.

### Bivariate GWAS results

**Fig. 1:**
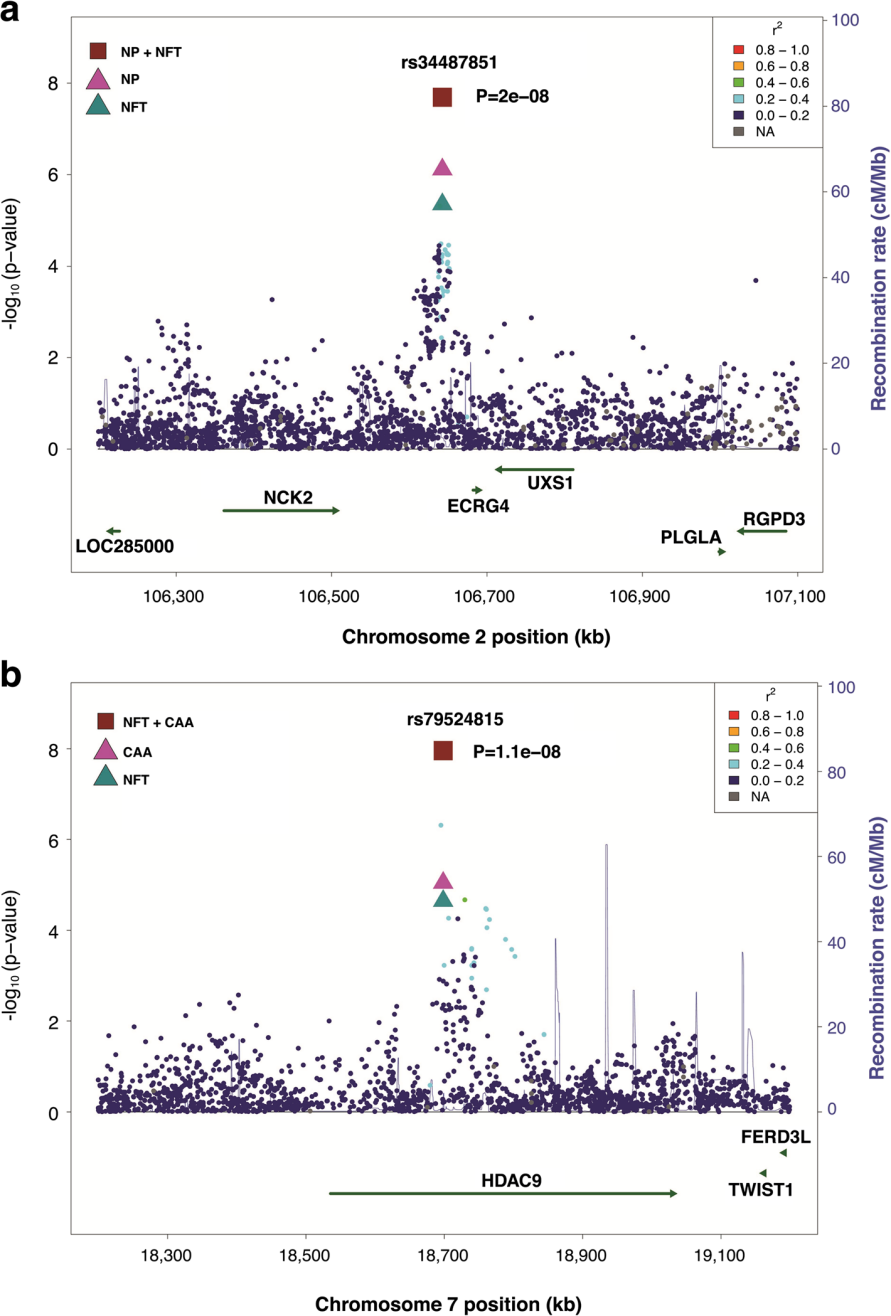
Regional association plots of (**a**) *C2orf40* from the joint model of neuritic plaque (NP) and neurofibrillary tangles (NFT) and (**b**) *HDAC9* from the joint model of NFT and cerebral amyloid angiopathy (CAA)

There was no inflation in *P* values for the GWAS of the three neuropathological traits analyzed individually (genomic control parameter, λ = 1.00, 1.01, and 0.96 for NP, NFT, and CAA, respectively) or as joint outcomes (Additional file 1: Figure S1). Results of the pleiotropy GWAS are shown in Additional file 1: Figure S2. As reported previously, with the exception of *APOE*, only 15 of 25 previously known AD loci attained at least a nominal association with NP, NFT, or CAA [6]. Three of the previously established AD loci—*BIN1*, *HLA* region, and *PICALM*—were moderately associated (*P* < 10^−4^) in the pleiotropy analysis for NP and NFT at a significance level of at least one order of magnitude smaller compared with the results from univariate analyses (Additional file 1: Table S2). Two novel GWS associations were detected in the pleiotropy analyses (Table 1, Fig. 1). rs34487851, an SNP located approximately 40 kb upstream of *C2orf40*, was associated with the joint model of NP and NFT (*P*_joint_ = 2.0 × 10^−8^). An intronic SNP in *HDAC9*, rs79524815, was associated with the joint model of NFT and CAA (*P*_joint_ = 1.1 × 10^−8^). The major allele *A* of rs34487851 and the minor allele *G* of rs79524815 are associated with increased NP and NFT and with increased NFT and the presence of CAA, respectively. Both of these findings were at least one order of magnitude more significant than for the univariate traits (Table 1) and were supported by evidence from multiple SNPs at those locations (Fig. 1, Additional file 1: Table S3).

**Table 1:** Genome-wide significant association (*P* < 5.0 × 10^−8^) of novel genes in the genome-wide pleiotropy analyses (joint models) of the three neuropathological traits neuritic plaque, neurofibrillary tangles, and cerebral amyloid angiopathy

						Univariate models								Joint models					
						AD status		NP		NFT		CAA		NP + NFT		NP + CAA		NFT + CAA	
Chromosome	SNP	Gene	EA	RA	EAF	β value (SE)	*P* value	β value (SE)	*P* value	β value (SE)	*P* value	β value (SE)	*P* value	Direction	*P* value	Direction	*P* value	Direction	*P* value
2	rs34487851	*ECRG4* ^a^	G	A	0.27	−0.42 (0.09)	5.8 × 10^−6^	−0.3 (0.06)	7.7 × 10^−7^	−0.25 (0.06)	4.5 × 10^−6^	−0.14 (0.08)	0.06	−	2.0 × 10^−8^	−	2.5 × 10^−6^	−	2.1 × 10^−5^
7	rs79524815	*HDAC9*	G	T	0.03	0.69 (0.31)	0.03	0.43 (0.19)	0.03	0.79 (0.19)	2.3 × 10^−5^	1.16 (0.26)	9.1 × 10^−6^	+	1.3 × 10^−4^	+	3.3 × 10^−6^	+	1.1 × 10^−8^

*Abbreviations: EA* Effect allele, *RA* Reference allele, *EAF* Effect allele frequency, *SNP* Single-nucleotide polymorphism, *AD* Alzheimer’s disease, *NP* Neuritic plaque, *NFT* Neurofibrillary tangles, *CAA* Cerebral amyloid angiopathy

^**a**^Also known as *C2orf40*

### Bivariate gene-based pleiotropy analysis results

Three contiguous novel genes on chromosome 2p25.3 (*TRAPPC12*, *TRAPPC12-AS1*, and *ADI1*) were associated with the joint model of NFT and CAA at a gene-wide significant level (*P* ≤ 2.0 × 10^−6^) (Table 2 and Additional file 1: Figure S3). Of note, one SNP in this region (rs35067331 in *TRAPPC12*) was associated with the NFT-CAA outcome at nearly the GWS level (*P*_joint_ = 5.8 × 10^−8^) (Additional file 1: Table S4).

**Table 2:** Gene-wide significant results (*P* < 2.7 × 10^−6^) from gene-based tests of pleiotropy single-nucleotide polymorphism association results

				Univariate gene-based tests			Pleiotropy gene-based tests		
Chromosome	Start	End	Gene	NP	NFT	CAA	NP + NFT	NP + CAA	NFT + CAA
2	3,383,446	3,483,342	*TRAPPC12*	0.09	4.0 × 10^−5^	0.5	0.01	0.07	2.0 × 10^−6^
2	3,485,013	3,486,180	*TRAPPC12-AS1*	0.002	3.9 × 10^−5^	5.0 × 10^−3^	1.6 × 10^−5^	2.1 × 10^−5^	< 1.0 × 10^−6^
2	3,501,690	3,523,350	*ADI1*	0.003	1.6 × 10^−5^	7.0 × 10^−4^	3.2 × 10^−5^	4.0 × 10^−6^	< 1.0 × 10^−6^

*Abbreviations: NP* Neuritic plaque, *NFT* Neurofibrillary tangles, *CAA* Cerebral amyloid angiopathy

Gene-based *P* values were computed through 1 million permutations, so the smallest *P* value is 1.0 × 10^−6^

### Trivariate GWAS and gene-based pleiotropy analysis results

We conducted trivariate GWAS and gene-based association analyses to identify genetic factors common to NP, NFT, and CAA. There was no evidence for genomic inflation (λ = 0.99) in the results from the trivariate model (Additional file 1: Figure S4). GWS association was observed only for *APOE* isoform SNP rs429358 (*P* = 2.1 × 10^−47^), whereas associations at *C2orf40*, *HDAC9*, and *TRAPPC12* were attenuated (Additional file 1: Table S5).

### eQTL analysis

We performed eQTL association analysis to examine whether the expression levels of the five GWS significant genes identified in the pleiotropy association tests differed between carriers and noncarriers of the risk alleles from those loci. Because information about the two GWS SNPs was not available in the GTEx Portal database or in the Mayo Clinic brain eGWAS, we analyzed proxy SNPs that are in high linkage disequilibrium (LD; *D′* ≥ 80) with the GWS SNPs. According to GTEx, rs34487851 proxy SNP rs1232803 is a *cis*-acting eQTL, and the major allele *A*, which is associated with higher NP and NFT, is also significantly associated with decreased expression of *C2orf40* in several tissues, including the esophagus (*P* = 3.5 × 10^−5^), transverse colon (*P* = 4.7 × 10^−4^), and tibial artery (*P* = 1.7 × 10^−3^), but not in any brain regions. In the Mayo Clinic brain eGWAS, proxy SNPs for rs34487851 were not *cis*-acting eQTLs for *C2orf40*. In GTEx, proxy SNPs for rs79524815 were not associated with the expression of *HDAC9*. However, in the brain eGWAS, the minor alleles of proxy SNPs for rs79524815, which are associated with higher NFT and CAA, were significantly associated with lower *HDAC9* levels in the CER (probe ID: ILMN_1803563; best eQTL, rs4721721; *P* = 0.003) but not in the TCX (Additional file 1: Table S3). According to GTEx, rs35067331 is a *cis*-acting eQTL, and its major allele *C*, which is associated with higher NFT and CAA, is significantly associated with increased expression of *TRAPPC12-AS1* in several brain regions (best *P* = 2.1 × 10^−7^ in cortex) and *ADI1* (*P* = 0.03) in the caudate nucleus, but not with differential expression of *TRAPPC12* in any brain regions (Additional file 1: Table S4). In the Mayo Clinic brain eGWAS data, rs35067331 and its proxy SNPs were not *cis*-acting eQTLs for *ADI1* or *TRAPPC12*. Unfortunately, information about *TRAPPC12-AS1* was unavailable in the brain eGWAS.

### Differential gene expression analysis

**Fig. 2:**
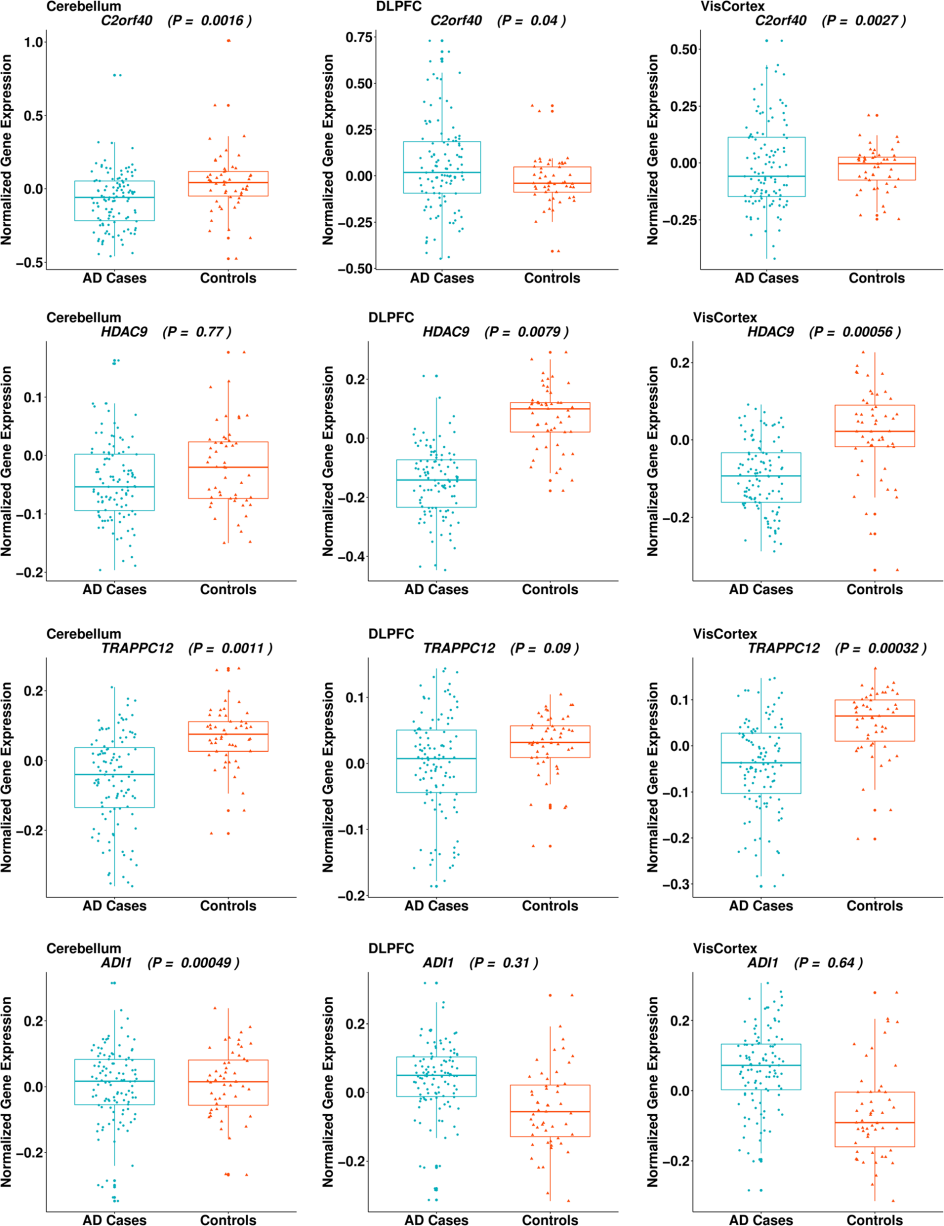
Box plots showing differential expression in microarray data [GEO:GSE44772] between AD cases and controls for *C2orf40*, *HDAC9*, *TRAPPC12*, and *ADI1* in the cerebellum (CER; left column), dorsolateral prefrontal cortex (DLPFC; middle column), and visual cortex (VCX; right column). AD Alzheimer’s disease

We investigated whether the expression levels of *C2orf40*, *HDAC9*, and *TRAPPC12*/*TRAPPC12-AS1*/*ADI1* differed in AD brains compared with non-AD control brains in the publicly available RNS-Seq and microarray datasets (Table 3 and Fig. 2). There were no significant differences in *C2orf40* expression between subjects with AD and control subjects in the TCX or CER in the Mayo Clinic RNA-Seq DGE profiling. However, *C2orf40* was significantly downregulated in subjects with AD compared with control subjects in the CER (*P* = 1.6 × 10^−3^), DLPFC (*P* = 0.04), and VCX (*P* = 2.7 × 10^−3^) in the microarray brain expression data. *HDAC9* was significantly downregulated in subjects with AD compared with control subjects in several brain regions, including the TCX (*P* = 1.5 × 10^−4^) and CER (*P* = 0.04) in the RNA-Seq profiling data and in the DLPFC (*P* = 7.9 × 10^−3^) and VCX (*P* = 5.6 × 10^−4^) in the microarray expression data. *ADI1* expression was downregulated in subjects with AD in the CER in the microarray data (*P* = 4.9 × 10^−4^). The RNA-Seq DGE profiling indicated that *TRAPPC12-AS1* expression was significantly increased in subjects with AD in the TCX (*P* = 1.3 × 10^−3^). In contrast, expression of *TRAPPC12* was significantly lower in subjects with AD than in control subjects in the TCX (*P* = 0.01) in the RNA-Seq and in the CER (*P* = 1.1 × 10^−3^) and VCX (*P* = 3.2 × 10^−4^) in the microarray data. Information about expression of *TRAPPC12-AS1* was not available in the microarray data. The majority of these nominally significant findings survived correction for multiple testing (*P* < 2.27 × 10^−3^).

**Table 3:** Results of differential gene expression analysis in brain

	RNA-Seq				Microarray					
	CER		TCX		CER		DLPFC		VCX	
Gene	β value (SE)	P value	β value (SE)	*P* value	β value (SE)	*P* value	β value (SE)	*P* value	β value (SE)	*P* value
*ECRG4* ^a^	0.06 (0.20)	0.77	0.19 (0.24)	0.43	−0.18 (0.05)	**1.6 × 10** ^**−3**^	−0.12 (0.06)	0.04	−0.12 (0.04)	2.7 × 10^−3^
*HDAC9*	−0.24 (0.12)	0.04	−0.31 (0.08)	**1.5 × 10** ^**−4**^	−0.01 (0.02)	0.77	−0.09 (0.03)	7.9 × 10^−3^	−0.06 (0.02)	**5.6 × 10** ^**−4**^
*TRAPPC12*	−0.05 (0.06)	0.35	−0.13 (0.05)	0.01	−0.09 (0.03)	**1.1 × 10** ^**−3**^	−0.03 (0.02)	0.09	−0.08 (0.02)	**3.2 × 10** ^**−4**^
*TRAPPC12-AS1*	0.22 (0.12)	0.06	0.59 (0.18)	**1.3 × 10** ^**−3**^	–	–	–	–	–	–
*ADI1*	−0.10 (0.08)	0.19	−0.07 (0.08)	0.36	−0.10 (0.03)	**4.9 × 10** ^**−4**^	−0.03 (0.03)	0.31	−0.01 (0.03)	0.64

*Abbreviations: CER* Cerebellum, *TCX* Temporal cortex, *DLPFC* Dorsolateral prefrontal cortex, *CER* Cerebellum, *TCX* Temporal cortex, *DLPFC* Dorsolateral prefrontal cortex, *VCX* Visual cortex

Results were obtained from analyses of RNA-Seq data in the Synapse database (https://www.synapse.org; [17]) and microarray data in the Gene Expression Omnibus database [GEO:GSE44771]. Negative β value indicates lower level of gene expression in AD cases compared with controls and vice versa. Results that remained significant after multiple test correction (*P* = 0.05/22 = 2.27 × 10^−3^) are highlighted in **bold**

^a^Also known as *C2orf40*

To contextualize our findings, we evaluated DGE among AD cases and controls for 26 previously established AD genes (Additional file 1: Table S6) [19–21]. With notable exceptions of *EPHA1* (*P* = 2.8 × 10^−7^) and *SLC24A4* (*P* = 7.0 × 10^−5^) in CER in the RNA-Seq data and *ABCA7* in DLPFC (*P* = 2.5 × 10^−7^) and VCX (*P* = 4.9 × 10^−7^) in the microarray data, none of the results for the other 23 genes were significant after correcting for 127 tests (*P* < 3.94 × 10^−4^).

## Discussion

A previous GWAS of neuropathologic traits including NP, NFT, and CAA identified GWS associations with *APOE* only [6]. Our pleiotropy analysis of all pairwise combinations of these traits identified GWS associations with *APOE* and three regions not previously reported with any neuropathologic traits or AD risk, including *C2orf40* for the joint model comprising NP and NFT, as well as *HDAC9* and *TRAPPC12/TRAPPC12-AS1/ADI1* for the joint model comprising NFT and CAA. Our DGE study found that *HDAC9* is significantly downregulated in several brain regions in subjects with AD compared with control subjects. Moreover, we observed that the *G* allele of *HDAC9* SNP rs79524815 is associated with a higher level of the joint outcome of NFT and CAA, and proxy SNPs for rs79524815 (which are suggestively associated with the joint outcome of NFT and CAA) are associated with decreased *HDAC9* expression in subjects with AD (Additional file 1: Table S3). The pleiotropy analysis also revealed that 4 (*BIN1*, *HLA*, *PICALM*, and *APOE*) of the 25 previously reported GWS AD risk loci [7, 19–21] were at least one order of magnitude more significantly associated with the joint model of NP + NFT than each of these traits analyzed separately, suggesting that these genes are involved in pathways leading to both plaques and tangles [22–25].

It is notable that pleiotropy analyses for the model including NP, NFT, and CAA did not yield any GWS findings. Moreover, the GWS associations identified in the bivariate models were attenuated in the trivariate model. These results suggest that the mechanisms or pathways underlying the bivariate associations probably do not encompass all three traits, and this conclusion may generalize genome-wide.

*C2orf40*, also known as *esophageal cancer-related gene 4* (*ECRG4*), is a tumor suppressor gene [26] that encodes a peptide hormone that is involved in NFT formation in transgenic mice [27], senescence of precursor cells in the CNS during aging [28], and activation of microglia and peripheral mononuclear leukocytes [29]. We observed that rs34487851 allele *A* is associated with higher NP and NFT and lower expression of *ERCG4,* albeit not in the brain. We also found that *ERCG4* expression was significantly lower in AD cases than in controls in several brain regions. Abnormal downregulation of *C2orf40* was previously reported in brain injury [30] as well as in several cancer cell types [31, 32].

*HDAC9* encodes a member of class IIa histone genes that deacetylate histones, thereby remodeling chromatin structure and controlling gene expression [33, 34] that has previously been linked to epigenetic mechanisms [35] and memory loss [36] in AD and also has been proposed as a possible therapeutic target [37–39]. GWS association of ischemic stroke with an *HDAC9* variant was identified by GWAS [40]. Structural variants including deletions and copy number variants in *HDAC9* have been identified in patients with schizophrenia and patients with autism [41, 42]. *MEF2C*, one of the well-established AD risk loci [21], stimulates *HDAC9* expression, but *HDAC9* suppresses *MEF2C* transcription, resulting in a negative feedback loop [43]. In a previously reported coexpression network study in AD and control brains, *HDAC9* and *MEF2C* were clustered together in the top fourth module ranked by relevance to AD pathology, and expression of *HDAC9* and *MEF2C* was inversely correlated with Braak stage (*HDAC9*, *r* = −0.71; *MEF2C*, *r* = −0.65) and frontal atrophy (*HDAC9*, *r* = −0.57, *MEF2C*, *r* = −0.51) [18]. These findings are consistent with our observation that *HDAC9* expression is reduced in subjects with AD and in the subjects with *HDAC9* SNP alleles associated with higher NFT and CAA. Decreased *HDAC9* expression has also been linked to increased neuronal apoptosis [44, 45]. Collectively, findings from our and other studies indicate that *MEF2C* and *HDAC9* may participate in a pathway leading to NFT formation and brain atrophy.

Gene-based analyses identified significant associations with three adjacent loci—*TRAPPC12*, *TRAPPC12-AS1*, and *ADI1*—in a gene-rich region near the end of the short arm of chromosome 2p. *ADI1*, encoding acireductone dioxygenase 1, is involved in methionine salvage and prostate cancer [46] and has no known relationship to AD. *TRAPPC12* is a subunit of a trafficking protein particle complex that has a role in vesicle trafficking in endoplasmic reticulum (ER) to Golgi [47]. *TRAPPC12-AS1* is an antisense (noncoding) RNA that contains a 1168 transcript from *TRAPPC12*. We previously established that regulation of vesicular trafficking in the ER to Golgi by several VPS10 receptor domain receptor genes, including *SORL1,* and by other genes encoding members of the retromer complex is an important pathway leading to AD [48–50]. Of the genes in this region, only *TRAPPC12-AS1* showed a pattern of expression in subjects with AD and control subjects that is consistent with the effect direction of the *TRAPPC12* rs35067331 allele’s influence on NFT and CAA. It should be noted that *TRAPPC12* expression was significantly lower in AD cases than in control subjects in the TCX (*P* = 0.01) in the RNA-Seq data and the CER (*P* = 1.1 × 10^−3^) and VCX (*P* = 3.2 × 10^−4^) in the microarray data, which could be due to negative feedback by the antisense *TRAPPC12-AS1* transcript [51].

Our study has several potential caveats. The GWS associations identified in the pleiotropy analysis have moderate supportive evidence for association from other SNPs under the association peaks, probably because of low LD with the top SNPs. The two GWS SNPs near *C2orf40* and *HDAC9* were not associated with AD risk in one of the largest GWASs for AD (rs34487851, *P* = 0.07; rs79524815, *P* = 0.73) [19, 21]. However, approximately 87% of the autopsy samples used in this pleiotropy analysis (as well as in the *Beecham et al.* study [6]) were from patients with AD. This may indicate that our findings are more relevant with neuropathological progression after onset of AD clinical symptoms. Alternatively, because our study was focused on endophenotypes that might be more proximal than disease diagnosis to effects of the genetic variants [52, 53], our analyses might have more power to detect those novel associations. Finally, to our knowledge, additional large late-onset AD cohorts with neuropathological and genotype data are not currently available for replication of our association findings. Therefore, validation of the role of these loci in AD will likely require experimental evidence.

## Conclusions

Our findings suggest that genome-wide pleiotropy analysis is a useful approach to identifying novel genetic associations with complex diseases and their endophenotypes. Functional studies are needed to determine whether *C2orf40* or *HDAC9* is a plausible therapeutic target.

## Additional files

Additional file 1: Table S1. Sample characteristics. **Table S2.** Association *P* values of Alzheimer disease loci previously established by GWAS in univariate and pleiotropy association tests of neuropathological features. **Table S3.** Association of *cis*-eQTL for *HDAC9* in the Mayo Clinic brain expression genome-wide association study (eGWAS). rs79524815 was not available in the Mayo Clinic brain eGWAS, so proxy SNPs that are in LD (D′ > 0.90) with rs79524815 were used for the eQTL test with *HDAC9* expression. **Table S4.** Association of expression of SNPs for *TRAPPC12-AS1* and *ADI1* with neuropathological traits and gene expression in the GTEx portal database. **Table S5.** Association results from the trivariate pleiotropy model of neuritic plaque (NP), neurofibrillary tangles (NFT), and cerebral amyloid angiopathy (CAA) for study-wide significant SNPs in the bivariate pleiotropy model. **Table S6.** Results of differential gene expression analysis by brain region among AD cases and controls for AD loci previously established by GWAS in RNA-Seq and microarray analysis. **Figure S1.** Quantile-quantile plots of observed (y-axis) vs. expected (x-axis) P values of all SNPs (black dots) and after excluding SNPs in *APOE* region (blue dots) for the pleiotropy analysis of (**a**) NP and NFT, (**b**) NP and CAA, and (**c**) NFT and CAA using the O’Brien method [10]. **Figure S2.** Manhattan plots showing genome-wide pleiotropy analyses of (**a**) NP and NFT, (**b**) NP and CAA, and (**c**) NFT and CAA using the O’Brien method [10]. Red dashed line represents the genome-wide significance threshold of *P* < 5.0 × 10^−−8^. Loci achieving genome-wide significance are highlighted in red, and known AD genes that attained at least a moderate significance level (*P* < 10^−−4^) are highlighted in gold. **Figure S3.** Regional association plots of genes, including *TRAPPC12*, *TRAPPC12-AS1*, and *ADI1,* on chromosome 2 from the joint model of NFT and CAA. **Figure S4.** Genome-wide trivariate pleiotropy analysis of NP, NFT, and CAA. (**a**) Quantile-quantile plot. (**b**) Manhattan plot. (DOCX 1028 kb)

## References

[1] Jorm AF, Jolley D (1998). The incidence of dementia: a meta-analysis. Neurology.

[2] Corrada MM, Brookmeyer R, Paganini-Hill A, Berlau D, Kawas CH (2010). Dementia incidence continues to increase with age in the oldest old: the 90+ study. Ann Neurol.

[3] Shoji M, Golde TE, Ghiso J, Cheung TT, Estus S, Shaffer LM, Cai XD, McKay DM, Tintner R, Frangione B (1992). Production of the Alzheimer amyloid β protein by normal proteolytic processing. Science.

[4] Trojanowski JQ, Schmidt ML, Shin RW, Bramblett GT, Rao D, Lee VM (1993). Altered tau and neurofilament proteins in neuro-degenerative diseases: diagnostic implications for Alzheimer’s disease and Lewy body dementias. Brain Pathol.

[5] Love S (2004). Contribution of cerebral amyloid angiopathy to Alzheimer’s disease. J Neurol Neurosurg Psychiatry.

[6] Beecham GW, Hamilton K, Naj AC, Martin ER, Huentelman M, Myers AJ, Corneveaux JJ, Hardy J, Vonsattel JP, Younkin SG (2014). Genome-wide association meta-analysis of neuropathologic features of Alzheimer’s disease and related dementias. PLoS Genet.

[7] Naj AC, Jun G, Beecham GW, Wang LS, Vardarajan BN, Buros J, Gallins PJ, Buxbaum JD, Jarvik GP, Crane PK (2011). Common variants at *MS4A4*/*MS4A6E*, *CD2AP*, *CD33* and *EPHA1* are associated with late-onset Alzheimer’s disease. Nat Genet.

[8] Mirra SS, Hart MN, Terry RD (1993). Making the diagnosis of Alzheimer’s disease: a primer for practicing pathologists. Arch Pathol Lab Med.

[9] Braak H, Braak E (1991). Neuropathological stageing of Alzheimer-related changes. Acta Neuropathol.

[10] O’Brien PC (1984). Procedures for comparing samples with multiple endpoints. Biometrics.

[11] Yang Q, Wu H, Guo CY, Fox CS (2010). Analyze multivariate phenotypes in genetic association studies by combining univariate association tests. Genet Epidemiol.

[12] Yang Q, Wang Y (2012). Methods for analyzing multivariate phenotypes in genetic association studies. J Probab Stat.

[13] Liu JZ, McRae AF, Nyholt DR, Medland SE, Wray NR, Brown KM, Investigators AMFS, Hayward NK, Montgomery GW, Visscher PM (2010). A versatile gene-based test for genome-wide association studies. Am J Hum Genet.

[14] GTEx Consortium (2015). The Genotype-Tissue Expression (GTEx) pilot analysis: multitissue gene regulation in humans. Science.

[15] Zou F, Chai HS, Younkin CS, Allen M, Crook J, Pankratz VS, Carrasquillo MM, Rowley CN, Nair AA, Middha S (2012). Brain expression genome-wide association study (eGWAS) identifies human disease-associated variants. PLoS Genet.

[16] Carrasquillo MM, Zou F, Pankratz VS, Wilcox SL, Ma L, Walker LP, Younkin SG, Younkin CS, Younkin LH, Bisceglio GD (2009). Genetic variation in *PCDH11X* is associated with susceptibility to late-onset Alzheimer’s disease. Nat Genet.

[17] Allen M, Carrasquillo MM, Funk C, Heavner BD, Zou F, Younkin CS, Burgess JD, Chai HS, Crook J, Eddy JA (2016). Human whole genome genotype and transcriptome data for Alzheimer’s and other neurodegenerative diseases. Sci Data.

[18] Zhang B, Gaiteri C, Bodea LG, Wang Z, McElwee J, Podtelezhnikov AA, Zhang C, Xie T, Tran L, Dobrin R (2013). Integrated systems approach identifies genetic nodes and networks in late-onset Alzheimer’s disease. Cell.

[19] Jun G, Ibrahim-Verbaas CA, Vronskaya M, Lambert JC, Chung J, Naj AC, Kunkle BW, Wang LS, Bis JC, Bellenguez C (2016). A novel Alzheimer disease locus located near the gene encoding tau protein. Mol Psychiatry.

[20] Jun GR, Chung J, Mez J, Barber R, Beecham GW, Bennett DA, Buxbaum JD, Byrd GS, Carrasquillo MM, Crane PK (2017). Transethnic genome-wide scan identifies novel Alzheimer’s disease loci. Alzheimers Dement.

[21] Lambert JC, Ibrahim-Verbaas CA, Harold D, Naj AC, Sims R, Bellenguez C, DeStafano AL, Bis JC, Beecham GW, Grenier-Boley B (2013). Meta-analysis of 74,046 individuals identifies 11 new susceptibility loci for Alzheimer’s disease. Nat Genet.

[22] Liu CC, Kanekiyo T, Xu H, Bu G (2013). Apolipoprotein E and Alzheimer disease: risk, mechanisms and therapy. Nat Rev Neurol.

[23] Minett T, Classey J, Matthews FE, Fahrenhold M, Taga M, Brayne C, Ince PG, Nicoll JA, Boche D, MRC CFAS (2016). Microglial immunophenotype in dementia with Alzheimer’s pathology. J Neuroinflammation.

[24] Rosenthal SL, Kamboh MI (2014). Late-onset Alzheimer’s disease genes and the potentially implicated pathways. Curr Genet Med Rep.

[25] Tan MS, Yu JT, Tan L (2013). Bridging integrator 1 (BIN1): form, function, and Alzheimer’s disease. Trends Mol Med.

[26] Li LW, Li YY, Li XY, Zhang CP, Zhou Y, Lu SH (2011). A novel tumor suppressor gene ECRG4 interacts directly with TMPRSS11A (ECRG1) to inhibit cancer cell growth in esophageal carcinoma. BMC Cancer.

[27] Woo JM, Park SJ, Kang HI, Kim BG, Shim SB, Jee SW, Lee SH, Sin JS, Bae CJ, Jang MK (2010). Characterization of changes in global gene expression in the brain of neuron-specific enolase/human Tau23 transgenic mice in response to overexpression of Tau protein. Int J Mol Med.

[28] Kujuro Y, Suzuki N, Kondo T (2010). Esophageal cancer-related gene 4 is a secreted inducer of cell senescence expressed by aged CNS precursor cells. Proc Natl Acad Sci U S A.

[29] Podvin S, Miller MC, Rossi R, Chukwueke J, Donahue JE, Johanson CE, Baird A, Stopa EG (2016). The orphan C2orf40 gene is a neuroimmune factor in Alzheimer’s disease. JSM Alzheimers Dis Relat Dement.

[30] Podvin S, Gonzalez AM, Miller MC, Dang X, Botfield H, Donahue JE, Kurabi A, Boissaud-Cooke M, Rossi R, Leadbeater WE (2011). Esophageal cancer related gene-4 is a choroid plexus-derived injury response gene: evidence for a biphasic response in early and late brain injury. PLoS One.

[31] Li LW, Yu XY, Yang Y, Zhang CP, Guo LP, Lu SH (2009). Expression of esophageal cancer related gene 4 (ECRG4), a novel tumor suppressor gene, in esophageal cancer and its inhibitory effect on the tumor growth in vitro and in vivo. Int J Cancer.

[32] Mori Y, Ishiguro H, Kuwabara Y, Kimura M, Mitsui A, Kurehara H, Mori R, Tomoda K, Ogawa R, Katada T (2007). Expression of ECRG4 is an independent prognostic factor for poor survival in patients with esophageal squamous cell carcinoma. Oncol Rep.

[33] Strahl BD, Allis CD (2000). The language of covalent histone modifications. Nature.

[34] Sugo N, Oshiro H, Takemura M, Kobayashi T, Kohno Y, Uesaka N, Song WJ, Yamamoto N (2010). Nucleocytoplasmic translocation of HDAC9 regulates gene expression and dendritic growth in developing cortical neurons. Eur J Neurosci.

[35] Millan MJ (2014). The epigenetic dimension of Alzheimer’s disease: causal, consequence, or curiosity?. Dialogues Clin Neurosci.

[36] Agis-Balboa RC, Pavelka Z, Kerimoglu C, Fischer A (2013). Loss of HDAC5 impairs memory function: implications for Alzheimer’s disease. J Alzheimers Dis.

[37] Fischer A (2014). Targeting histone-modifications in Alzheimer’s disease: what is the evidence that this is a promising therapeutic avenue?. Neuropharmacology.

[38] Cuadrado-Tejedor M, Oyarzabal J, Lucas MP, Franco R, Garcia-Osta A (2013). Epigenetic drugs in Alzheimer’s disease. Biomol Concepts.

[39] Benito E, Urbanke H, Ramachandran B, Barth J, Halder R, Awasthi A, Jain G, Capece V, Burkhardt S, Navarro-Sala M (2015). HDAC inhibitor-dependent transcriptome and memory reinstatement in cognitive decline models. J Clin Invest.

[40] Bellenguez C, Bevan S, Gschwendtner A, Spencer CC, Burgess AI, Pirinen M, Jackson CA, Traylor M, International Stroke Genetics Consortium (ISGC), Wellcome Trust Case Control Consortium 2 (WTCC2) (2012). Genome-wide association study identifies a variant in *HDAC9* associated with large vessel ischemic stroke. Nat Genet.

[41] Pinto D, Delaby E, Merico D, Barbosa M, Merikangas A, Klei L, Thiruvahindrapuram B, Xu X, Ziman R, Wang Z (2014). Convergence of genes and cellular pathways dysregulated in autism spectrum disorders. Am J Hum Genet.

[42] Tam GW, van de Lagemaat LN, Redon R, Strathdee KE, Croning MD, Malloy MP, Muir WJ, Pickard BS, Deary IJ, Blackwood DH (2010). Confirmed rare copy number variants implicate novel genes in schizophrenia. Biochem Soc Trans.

[43] Haberland M, Arnold MA, McAnally J, Phan D, Kim Y, Olson EN (2007). Regulation of *HDAC9* gene expression by MEF2 establishes a negative-feedback loop in the transcriptional circuitry of muscle differentiation. Mol Cell Biol.

[44] Morrison BE, Majdzadeh N, Zhang X, Lyles A, Bassel-Duby R, Olson EN, D’Mello SR (2006). Neuroprotection by histone deacetylase-related protein. Mol Cell Biol.

[45] Salian-Mehta S, Xu M, McKinsey TA, Tobet S, Wierman ME (2015). Novel interaction of class IIb histone deacetylase 6 (HDAC6) with class IIa HDAC9 controls gonadotropin releasing hormone (GnRH) neuronal cell survival and movement. J Biol Chem.

[46] Oram SW, Ai J, Pagani GM, Hitchens MR, Stern JA, Eggener S, Pins M, Xiao W, Cai X, Haleem R (2007). Expression and function of the human androgen-responsive gene *ADI1* in prostate cancer. Neoplasia.

[47] Scrivens PJ, Noueihed B, Shahrzad N, Hul S, Brunet S, Sacher M (2011). C4orf41 and TTC-15 are mammalian TRAPP components with a role at an early stage in ER-to-Golgi trafficking. Mol Biol Cell.

[48] Reitz C, Jun G, Naj A, Rajbhandary R, Vardarajan BN, Wang LS, Valladares O, Lin CF, Larson EB, Graff-Radford NR (2013). Variants in the ATP-binding cassette transporter (*ABCA7*), apolipoprotein E ε4, and the risk of late-onset Alzheimer disease in African Americans. JAMA.

[49] Rogaeva E, Meng Y, Lee JH, Gu Y, Kawarai T, Zou F, Katayama T, Baldwin CT, Cheng R, Hasegawa H (2007). The neuronal sortilin-related receptor SORL1 is genetically associated with Alzheimer disease. Nat Genet.

[50] Vardarajan BN, Bruesegem SY, Harbour ME, Inzelberg R, Friedland R, St George-Hyslop P, Seaman MN, Farrer LA (2012). Identification of Alzheimer disease-associated variants in genes that regulate retromer function. Neurobiol Aging.

[51] Pelechano V, Steinmetz LM (2013). Gene regulation by antisense transcription. Nat Rev Genet.

[52] Cannon TD, Keller MC (2006). Endophenotypes in the genetic analyses of mental disorders. Annu Rev Clin Psychol.

[53] Flint J, Munafo MR (2007). The endophenotype concept in psychiatric genetics. Psychol Med.

