# Supplementary material for: Genome-wide pleiotropy analysis of neuropathological traits related to Alzheimer’s disease
Source: Alzheimers Res Ther. 2018 Feb 20;10:22. doi: 10.1186/s13195-018-0349-z (PMC5819208; doi:10.1186/s13195-018-0349-z)
Supplement: Supplementary file 1 — Sample characteristics. Table S2. Association P values of Alzheimer disease loci previously established by GWAS in univariate and pleiotropy association tests of neuropathological features. Table S3. Association of cis-eQTL for HDAC9 in the Mayo Clinic brain expression genome-wide association study (eGWAS). rs79524815 was not available in the Mayo Clinic brain eGWAS, so proxy SNPs that are in LD (D′ > 0.90) with rs79524815 were used for the eQTL test with HDAC9 expression. Table S4. Association of expression of SNPs for TRAPPC12-AS1 and ADI1 with neuropathological traits and gene expression in the GTEx portal database. Table S5. Association results from the trivariate pleiotropy model of neuritic plaque (NP), neurofibrillary tangles (NFT), and cerebral amyloid angiopathy (CAA) for study-wide significant SNPs in the bivariate pleiotropy model. Table S6. Results of differential gene expression analysis by brain region among AD cases and controls for AD loci previously established by GWAS in RNA-Seq and microarray analysis. Figure S1. Quantile-quantile plots of observed (y-axis) vs. expected (x-axis) P values of all SNPs (black dots) and after excluding SNPs in APOE region (blue dots) for the pleiotropy analysis of (a) NP and NFT, (b) NP and CAA, and (c) NFT and CAA using the O’Brien method [10]. Figure S2. Manhattan plots showing genome-wide pleiotropy analyses of (a) NP and NFT, (b) NP and CAA, and (c) NFT and CAA using the O’Brien method [10]. Red dashed line represents the genome-wide significance threshold of P < 5.0 × 10−−8. Loci achieving genome-wide significance are highlighted in red, and known AD genes that attained at least a moderate significance level (P < 10−−4) are highlighted in gold. Figure S3. Regional association plots of genes, including TRAPPC12, TRAPPC12-AS1, and ADI1, on chromosome 2 from the joint model of NFT and CAA. Figure S4. Genome-wide trivariate pleiotropy analysis of NP, NFT, and CAA. (a) Quantile-quantile plot. (b) Manhattan plot. (DOCX [file 13195_2018_349_MOESM1_ESM.docx]

**Additional file 1**

**Table S1.** Sample characteristics

|  | **AD Cases** | **Controls** |
| --- | --- | --- |
| N | 3,135 | 463 |
| Female (%) | 1,742 (55.6%) | 219 (47.3%) |
| Age (SD) | 73.9 (7.9) | 78 (8.8) |
| *APOE* ε4 carriers | 1,984 | 83 |
| **Neuritic plaque** |  |  |
| None | 0 | 354 |
| Sparse | 0 | 109 |
| Moderate | 507 | 0 |
| Frequent | 2,628 | 0 |
| **Neurofibrillary tangles** |  |  |
| 0 | 0 | 70 |
| I-II | 0 | 393 |
| III-IV | 671 | 0 |
| V-VI | 2,464 | 0 |
| **Cerebral amyloid angiopathy** |  |  |
| any | 703 | 130 |
| absence | 1,531 | 39 |

**Table S2.** Association *P*-values of Alzheimer disease loci previously established by GWAS in univariate and pleiotropy association tests of neuropathological features.

|  |  |  |  |  | **Univariate analysis** | | | | | | | | | | |  | **Bivariate analysis** | | | | | | | |
| --- | --- | --- | --- | --- | --- | --- | --- | --- | --- | --- | --- | --- | --- | --- | --- | --- | --- | --- | --- | --- | --- | --- | --- | --- |
|  |  |  |  |  | **NP** | | |  | **NFT** | | |  | **CAA** | | |  | **NP+NFT** | |  | **NP+CAA** | |  | **NFT+CAA** | |
| **CHR** | **SNP** | **MA** | **MAF** | **Gene** | **β** | **SE** | **P** |  | **β** | **SE** | **P** |  | **β** | **SE** | **P** |  | **Dir** | **P** |  | **Dir** | **P** |  | **Dir** | **P** |
| 1 | rs6656401 | A | 0.20 | *CR1* | 0.02 | 0.06 | 0.76 |  | 0.09 | 0.06 | 0.10 |  | 0.03 | 0.08 | 0.67 |  | + | 0.25 |  | + | 0.61 |  | + | 0.17 |
| 2 | rs6733839 | T | 0.43 | *BIN1* | 0.24 | 0.06 | 9.2x10^-6^ |  | 0.22 | 0.05 | 7.8x10^-6^ |  | 0.02 | 0.06 | 0.71 |  | + | 1.6x10^-7^ |  | + | 9.8x10^-4^ |  | + | 1.5x10^-3^ |
| 2 | rs35349669 | T | 0.50 | *INPP5D* | -0.02 | 0.05 | 0.70 |  | 0.00 | 0.05 | 0.95 |  | -0.03 | 0.06 | 0.67 |  | - | 0.80 |  | - | 0.58 |  | - | 0.75 |
| 5 | rs190982 | G | 0.41 | *MEF2C* | -0.10 | 0.05 | 0.06 |  | -0.10 | 0.05 | 0.03 |  | -0.02 | 0.06 | 0.80 |  | - | 0.02 |  | - | 0.15 |  | - | 0.11 |
| 5 | rs11168036 | T | 0.49 | *HBEGF* | 0.01 | 0.05 | 0.85 |  | 0.03 | 0.05 | 0.56 |  | 0.13 | 0.07 | 0.04 |  | + | 0.65 |  | + | 0.12 |  | + | 0.08 |
| 6 | rs113788164 | C | 0.06 | *HLA region* | -0.61 | 0.16 | 1.5x10^-4^ |  | -0.53 | 0.13 | 8.5x10^-5^ |  | 0.01 | 0.19 | 0.95 |  | - | 5.5x10^-6^ |  | - | 0.01 |  | - | 0.01 |
| 6 | rs10948363 | G | 0.27 | *CD2AP* | 0.08 | 0.06 | 0.14 |  | 0.06 | 0.05 | 0.22 |  | -0.02 | 0.07 | 0.81 |  | + | 0.11 |  | + | 0.39 |  | + | 0.52 |
| 7 | rs2718058 | G | 0.36 | *NME8* | -0.06 | 0.05 | 0.26 |  | 0.06 | 0.05 | 0.19 |  | 0.04 | 0.06 | 0.53 |  | + | 0.91 |  | - | 0.74 |  | + | 0.20 |
| 7 | rs1476679 | C | 0.28 | *ZCWPW1* | -0.09 | 0.05 | 0.09 |  | -0.10 | 0.05 | 0.04 |  | 0.07 | 0.07 | 0.31 |  | - | 0.03 |  | - | 0.65 |  | - | 0.49 |
| 7 | rs11771145 | A | 0.33 | *EPHA1* | -0.06 | 0.05 | 0.22 |  | -0.11 | 0.05 | 0.03 |  | 0.07 | 0.06 | 0.27 |  | - | 0.04 |  | - | 0.96 |  | - | 0.47 |
| 8 | rs28834970 | C | 0.37 | *PTK2B* | 0.08 | 0.05 | 0.12 |  | 0.02 | 0.05 | 0.60 |  | -0.10 | 0.06 | 0.11 |  | + | 0.22 |  | - | 0.96 |  | - | 0.48 |
| 8 | rs9331896 | C | 0.38 | *CLU* | -0.08 | 0.05 | 0.15 |  | -0.06 | 0.05 | 0.17 |  | -0.06 | 0.07 | 0.38 |  | - | 0.10 |  | - | 0.11 |  | - | 0.14 |
| 10 | rs7920721 | G | 0.39 | *USP6NL* | -0.04 | 0.05 | 0.48 |  | 0.04 | 0.05 | 0.43 |  | 0.05 | 0.06 | 0.39 |  | + | 0.95 |  | + | 0.90 |  | + | 0.27 |
| 11 | rs10838725 | C | 0.32 | *CELF1* | 0.01 | 0.05 | 0.92 |  | 0.03 | 0.05 | 0.57 |  | -0.06 | 0.06 | 0.35 |  | + | 0.69 |  | - | 0.56 |  | - | 0.81 |
| 11 | rs983392 | G | 0.40 | *MS4A region* | -0.09 | 0.05 | 0.07 |  | -0.09 | 0.05 | 0.06 |  | -0.03 | 0.06 | 0.65 |  | - | 0.03 |  | - | 0.12 |  | - | 0.13 |
| 11 | rs10792832 | A | 0.35 | *PICALM* | -0.19 | 0.05 | 2.1x10^-4^ |  | -0.15 | 0.05 | 1.2x10^-3^ |  | -0.05 | 0.06 | 0.47 |  | - | 4.42x10^-5^ |  | - | 0.00 |  | - | 0.01 |
| 11 | rs11218343 | C | 0.04 | *SORL1* | -0.19 | 0.12 | 0.13 |  | -0.25 | 0.10 | 0.02 |  | . | . | . |  | - | 0.02 |  | - | 0.26 |  | - | 0.09 |
| 14 | rs17125944 | C | 0.09 | *FERMT2* | 0.19 | 0.09 | 0.03 |  | 0.08 | 0.08 | 0.27 |  | -0.10 | 0.10 | 0.36 |  | + | 0.05 |  | + | 0.39 |  | + | 0.91 |
| 14 | rs10498633 | T | 0.21 | *SLC24A4* | 0.02 | 0.06 | 0.75 |  | -0.06 | 0.05 | 0.22 |  | 0.00 | 0.07 | 0.96 |  | - | 0.59 |  | + | 0.85 |  | - | 0.40 |
| 17 | rs2732703 | G | 0.12 | *KANSL1* | 0.18 | 0.11 | 0.10 |  | 0.07 | 0.09 | 0.43 |  | 0.12 | 0.12 | 0.33 |  | + | 0.15 |  | + | 0.07 |  | + | 0.24 |
| 17 | rs116089788 | A | 0.02 | *BZRAP1* | -0.15 | 0.19 | 0.44 |  | -0.29 | 0.14 | 0.04 |  | -0.68 | 0.26 | 8.8x10^-3^ |  | - | 0.09 |  | - | 0.02 |  | - | 2.0x10^-3^ |
| 19 | rs4147929 | A | 0.19 | *ABCA7* | 0.14 | 0.07 | 0.04 |  | 0.13 | 0.06 | 0.03 |  | 0.06 | 0.08 | 0.47 |  | + | 0.01 |  | + | 0.05 |  | + | 0.06 |
| 19 | rs429358 | C | 0.25 | *APOE* | 0.98 | 0.08 | 1.3x10^-35^ |  | 0.81 | 0.07 | 1.9x10^-33^ |  | 0.61 | 0.09 | 1.9x10^-11^ |  | + | 3.4x10^-47^ |  | + | 4.8x10^-40^ |  | + | 5.8x10^-35^ |
| 19 | rs7412 | T | 0.06 | *APOE* | -0.43 | 0.15 | 3.4x10^-3^ |  | -0.32 | 0.14 | 0.02 |  | 0.04 | 0.18 | 0.83 |  | - | 1.8x10^-3^ |  | - | 0.06 |  | - | 0.16 |
| 19 | rs3865444 | A | 0.30 | *CD33* | -0.14 | 0.05 | 1.1x10^-2^ |  | -0.06 | 0.05 | 0.18 |  | -0.07 | 0.07 | 0.31 |  | - | 0.02 |  | - | 0.01 |  | - | 0.12 |
| 20 | rs7274581 | C | 0.08 | *CASS4* | -0.15 | 0.09 | 0.10 |  | -0.26 | 0.08 | 1.8x10^-3^ |  | 0.07 | 0.11 | 0.57 |  | - | 4.9x10^-3^ |  | - | 0.47 |  | - | 0.10 |

**Table S3.** Association of *cis*-eQTL for *HDAC9* in the Mayo Clinic brain expression genome-wide association study (eGWAS). Rs79524815 was not available in the Mayo Clinic brain eGWAS, so proxy SNPs that are LD (D` > 0.90) with rs79524815 were used to for eQTL test with *HDAC9* expressions.

|  |  |  |  |  |  |  |  |  | **Neuropathological Traits** | | | | | | | |  | **AD Status** | | | | | |
| --- | --- | --- | --- | --- | --- | --- | --- | --- | --- | --- | --- | --- | --- | --- | --- | --- | --- | --- | --- | --- | --- | --- | --- |
|  |  |  |  |  |  |  |  |  | **NFT** | | | **CAA** | | | **NFT+CAA** | |  | **AD** | | **Non-AD** | | **ALL**  **(AD + Non-AD)** | |
| **SNP** | **CH** | **EA** | **RA** | **EAF** |  | **r^2^** | **D`** |  | **β** | **Se** | **P** | **β** | **Se** | **P** | **Dir** | **P** |  | **β** | **P** | **β** | **P** | **β** | **P** |
| rs17348528 | 7 | C | T | 0.10 |  | 0.24 | 0.90 |  | 0.24 | 0.08 | 0.002 | 0.31 | 0.10 | 0.003 | + | 5.4x10^-5^ |  | -0.14 | 0.03 | -0.13 | 0.11 | -0.13 | 0.008 |
| rs4721719 | 7 | A | G | 0.11 |  | 0.24 | 0.90 |  | 0.20 | 0.07 | 0.005 | 0.27 | 0.10 | 0.006 | + | 2.5x10^-4^ |  | -0.11 | 0.08 | -0.14 | 0.06 | -0.13 | 0.007 |
| rs4721720 | 7 | A | G | 0.11 |  | 0.23 | 0.90 |  | 0.19 | 0.07 | 0.006 | 0.24 | 0.10 | 0.014 | + | 6.0x10^-4^ |  | -0.11 | 0.08 | -0.14 | 0.06 | -0.13 | 0.007 |
| rs4721721 | 7 | C | T | 0.12 |  | 0.23 | 0.95 |  | 0.16 | 0.07 | 0.021 | 0.24 | 0.09 | 0.009 | + | 1.1x10^-3^ |  | -0.09 | 0.17 | -0.19 | 0.01 | -0.14 | 0.003 |
| rs6960494 | 7 | G | A | 0.11 |  | 0.26 | 0.95 |  | 0.21 | 0.07 | 0.004 | 0.24 | 0.10 | 0.016 | + | 5.2x10^-4^ |  | -0.09 | 0.16 | -0.11 | 0.17 | -0.11 | 0.035 |

Brain region: Cerebellum

Probe ID: ILMN_1803563

EA: effect allele. RA: reference allele. EAF: effect allele frequency.

r^2^ and D` with rs79524815

Linear regression was used for eQTL association tests between expression levels (outcome) and imputed SNPs (predictor) after adjusting for age at death, gender, plate, RIN, (RIN-RIN_mean_)^2^, and *APOE* ε4 dosage. For the ALL sample, AD status as covariate was also included in the model.

Negative beta (β) for eQTL implies that carriers with effect allele have lower expression of *HDAC9* compared to non-carriers.

**Table S4.** Association of eSNP for *TRAPPC12-AS1* and *ADI1* with neuropathological traits and gene expression in the GTEx portal database.

|  |  |  |  |  | **Association of eSNP with Neuropathological Traits** | | | | | | | |  | **Association of eSNP with Expression by Brain Region** | | | |
| --- | --- | --- | --- | --- | --- | --- | --- | --- | --- | --- | --- | --- | --- | --- | --- | --- | --- |
|  |  |  |  |  | **NFT** | |  | **CAA** | |  | **NFT+CAA** | |  | **Gene** | **β (SE) *** | **P** | **Tissue** |
| **SNP** | **EA** | **RA** | **EAF** |  | **β (SE)** | **P** |  | **β (SE)** | **P** |  | **Dir** | **P** |  |  |  |  |  |
| rs35067331 | T | C | 0.27 |  | -0.25 (0.05) | 2.5x10^-7^ |  | -0.21 (0.07) | 2.0x10^-3^ |  | - | 5.8x10^-8^ |  | *ADI1* | -0.18 (0.08) | 0.03 | Caudate |
|  |  |  |  |  |  |  |  |  |  |  |  |  |  |  | -0.17 (0.08) | 0.05 | Hippocampus |
|  |  |  |  |  |  |  |  |  |  |  |  |  |  | *TRAPPC12-AS1* | -0.59 (0.10) | 2.1x10^-7^ | Cortex |
|  |  |  |  |  |  |  |  |  |  |  |  |  |  |  | -0.40 (0.10) | 2.7x10^-4^ | Nucleus accumbens |
|  |  |  |  |  |  |  |  |  |  |  |  |  |  |  | -0.42 (0.12) | 6.8x10^-4^ | Frontal Cortex |
|  |  |  |  |  |  |  |  |  |  |  |  |  |  |  | -0.39 (0.14) | 0.01 | Putamen |
|  |  |  |  |  |  |  |  |  |  |  |  |  |  |  | -0.35 (0.14) | 0.02 | Anterior cingulate cortex |
|  |  |  |  |  |  |  |  |  |  |  |  |  |  |  | -0.29 (0.13) | 0.03 | Hippocampus |
|  |  |  |  |  |  |  |  |  |  |  |  |  |  |  | -0.27 (0.12) | 0.03 | Caudate |

EA: effect allele. RA: reference allele. EAF: effect allele frequency; Dir = direction of effect

* Negative beta (β) indicates that carriers with effect allele have lower expression compared to non-carriers.

**Table S5.** Association results from the trivariate pleiotropy model of neuritic plaque (NP), neurofibrillary tangles (NFT), and cerebral amyloid angiopathy (CAA) for study-wide significant SNPs in the bivariate pleiotropy model.

|  |  |  |  |  |  |  | **Trivariate Model** | |
| --- | --- | --- | --- | --- | --- | --- | --- | --- |
|  |  |  |  |  |  |  | **NP + NFT + CAA** | |
| **CH** | **SNP** | **Gene** | **EA** | **RA** | **EAF** |  | **Dir** | **P** |
| 2 | rs34487851 | *ECRG4* * | G | A | 0.27 |  | - | 2.1x10^-7^ |
| 7 | rs79524815 | *HDAC9* | G | T | 0.03 |  | + | 1.1x10^-7^ |
| 2 | rs35067331 | *ADI1*  *TRAPPC12*  *TRAPPC12-AS1* | T | C | 0.27 |  | - | 5.5x10^-7^ |

***** gene also known as *C2orf40*

EA = effect allele, RA = reference allele, EAF = effect allele frequency.

**Table S6.** Results of differential gene expression analysis by brain region among Alzheimer disease cases and controls for AD loci previously established by GWAS in RNA-Seq and microarray.

|  | **RNA-Seq** | | | | | | |  | **Microarray** | | | | | | | | | | | |
| --- | --- | --- | --- | --- | --- | --- | --- | --- | --- | --- | --- | --- | --- | --- | --- | --- | --- | --- | --- | --- |
|  | **CER** | | |  | **TCX** | | |  |  | **CER** | | |  | **DLPFC** | | |  | **VCX** | | |
| **Gene** | **β** | **SE** | **P** |  | **β** | **SE** | **P** |  | **Probe ID** | **β** | **SE** | **P** |  | **β** | **SE** | **P** |  | **β** | **SE** | **P** |
| *CR1* | 0.40 | 0.26 | 0.12 |  | 0.06 | 0.30 | 0.84 |  | 10025912047 | 0.01 | 0.05 | 0.85 |  | -0.05 | 0.06 | 0.47 |  | 0.04 | 0.03 | 0.15 |
| *BIN1* | 0.00 | 0.05 | 0.95 |  | 0.01 | 0.10 | 0.92 |  | 10025906206 | 0.00 | 0.02 | 0.89 |  | 0.02 | 0.01 | 0.14 |  | 0.04 | 0.01 | 0.008 |
| *INPP5D* | -0.17 | 0.11 | 0.12 |  | -0.04 | 0.09 | 0.63 |  | 10023827621 | 0.02 | 0.02 | 0.35 |  | 0.06 | 0.02 | 0.01 |  | 0.03 | 0.02 | 0.22 |
| *MEF2C* | -0.09 | 0.09 | 0.32 |  | -0.01 | 0.16 | 0.97 |  | 10023809080 | 0.00 | 0.02 | 0.97 |  | -0.05 | 0.04 | 0.17 |  | -0.05 | 0.03 | 0.08 |
| *HBEGF* | -0.26 | 0.09 | 0.004 |  | -0.11 | 0.13 | 0.40 |  | 10023816362 | -0.04 | 0.02 | 0.08 |  | -0.03 | 0.02 | 0.26 |  | 0.01 | 0.02 | 0.7 |
| *HLA-DRB5* | -0.28 | 0.48 | 0.57 |  | 0.03 | 0.51 | 0.95 |  | 10025904809 | 0.15 | 0.1 | 0.13 |  | 0.2 | 0.13 | 0.13 |  | 0.15 | 0.11 | 0.16 |
| *HLA-DRB1* | -0.13 | 0.26 | 0.62 |  | -0.05 | 0.22 | 0.81 |  | 10023817834 | -0.05 | 0.05 | 0.34 |  | -0.02 | 0.06 | 0.81 |  | -0.04 | 0.06 | 0.51 |
| *CD2AP* | 0.10 | 0.05 | 0.05 |  | -0.03 | 0.06 | 0.59 |  | 10025908554 | 0.02 | 0.02 | 0.25 |  | -0.01 | 0.02 | 0.49 |  | 0.02 | 0.01 | 0.2 |
| *NME8* | -0.49 | 0.21 | 0.02 |  | -0.25 | 0.30 | 0.40 |  | . | . | . | . |  | . | . | . |  | . | . | . |
| *ZCWPW1* | -0.10 | 0.07 | 0.20 |  | -0.08 | 0.09 | 0.35 |  | 10025911117 | -0.04 | 0.02 | 0.02 |  | 0 | 0.02 | 0.83 |  | -0.02 | 0.02 | 0.36 |
| *EPHA1* | -0.38 | 0.07 | 2.8x10^-7^ |  | -0.07 | 0.08 | 0.41 |  | 10025909326 | 0.01 | 0.01 | 0.19 |  | 0.01 | 0.01 | 0.28 |  | 0.01 | 0.01 | 0.43 |
| *PTK2B* | -0.23 | 0.07 | 0.002 |  | -0.14 | 0.11 | 0.20 |  | 10025906234 | -0.05 | 0.03 | 0.04 |  | 0.07 | 0.03 | 0.005 |  | 0.01 | 0.03 | 0.64 |
| *CLU* | 0.25 | 0.14 | 0.07 |  | 0.36 | 0.12 | 0.004 |  | 10023824673 | -0.03 | 0.03 | 0.23 |  | 0 | 0.03 | 0.94 |  | 0.09 | 0.03 | 0.005 |
| *USP6NL* | -0.17 | 0.07 | 0.02 |  | -0.10 | 0.06 | 0.10 |  | 10025909277 | -0.04 | 0.03 | 0.08 |  | -0.03 | 0.02 | 0.10 |  | -0.07 | 0.02 | 0.001 |
| *CELF1* | 0.07 | 0.04 | 0.10 |  | 0.00 | 0.05 | 0.99 |  | . | . | . | . |  | . | . | . |  | . | . | . |
| *MS4A6A* | -0.14 | 0.20 | 0.48 |  | -0.14 | 0.12 | 0.26 |  | 10025904951 | 0.07 | 0.03 | 0.03 |  | 0.09 | 0.04 | 0.02 |  | 0.12 | 0.04 | 0.004 |
| *PICALM* | -0.07 | 0.04 | 0.09 |  | 0.09 | 0.05 | 0.09 |  | 10023808043 | 0.00 | 0.03 | 0.95 |  | -0.01 | 0.03 | 0.69 |  | 0.03 | 0.03 | 0.31 |
| *SORL1* | 0.12 | 0.06 | 0.04 |  | -0.04 | 0.08 | 0.65 |  | 10023811981 | -0.01 | 0.02 | 0.71 |  | -0.04 | 0.02 | 0.03 |  | -0.08 | 0.02 | 6.0x10^-4^ |
| *FERMT2* | 0.08 | 0.07 | 0.26 |  | 0.18 | 0.09 | 0.04 |  | . | . | . | . |  | . | . | . |  | . | . | . |
| *SLC24A4* | 0.90 | 0.22 | 7.0x10^-5^ |  | 0.18 | 0.13 | 0.17 |  | 10025913181 | 0.03 | 0.04 | 0.46 |  | 0.06 | 0.03 | 0.02 |  | 0.07 | 0.03 | 0.03 |
| *MAPT* | 0.06 | 0.04 | 0.10 |  | 0.03 | 0.04 | 0.43 |  | 10025904384 | 0.04 | 0.01 | 0.004 |  | 0.02 | 0.01 | 0.21 |  | 0.03 | 0.01 | 0.01 |
| *BZRAP1* | 0.24 | 0.07 | 0.001 |  | -0.14 | 0.17 | 0.42 |  | 10023808047 | 0.05 | 0.02 | 0.009 |  | 0.04 | 0.02 | 0.08 |  | 0.08 | 0.02 | 0.002 |
| *ABCA7* | -0.06 | 0.14 | 0.66 |  | 0.11 | 0.12 | 0.39 |  | 10025904236 | 0.04 | 0.02 | 0.09 |  | 0.1 | 0.02 | 2.5x10^-7^ |  | 0.12 | 0.02 | 4.9x10^-7^ |
| *APOE* | 0.01 | 0.16 | 0.96 |  | 0.13 | 0.15 | 0.38 |  | 10023813203 | -0.01 | 0.03 | 0.58 |  | -0.02 | 0.03 | 0.42 |  | 0.03 | 0.03 | 0.39 |
| *CD33* | -0.47 | 0.18 | 0.02 |  | -0.17 | 0.12 | 0.15 |  | 10023821464 | 0.00 | 0.02 | 0.91 |  | 0.00 | 0.02 | 0.96 |  | -0.02 | 0.02 | 0.23 |
| *CASS4* | 0.09 | 0.14 | 0.53 |  | -0.15 | 0.17 | 0.36 |  | . | . | . | . |  | . | . | . |  | . | . | . |

Results were obtained from analyses of RNA-Seq data in the Synapse database (<https://www.synapse.org>) and microarray data in the gene expression omnibus (GSE44771). Negative β indicates lower gene expression in AD cases compared with controls. CER = cerebellum; TCX = temporal cortex; DLPFC = dorsolateral prefrontal cortex; VCX = visual cortex.

**Figure S1.** Quantile-quantile plots of observed (y-axis) vs. expected (x-axis) P-values of all SNPs (black dots) and after excluding SNPs in *APOE* region (blue dots) for the pleiotropy analysis of (A) NP and NFT, (B) NP and CAA, and (C) NFT and CAA using the O’Brien method.


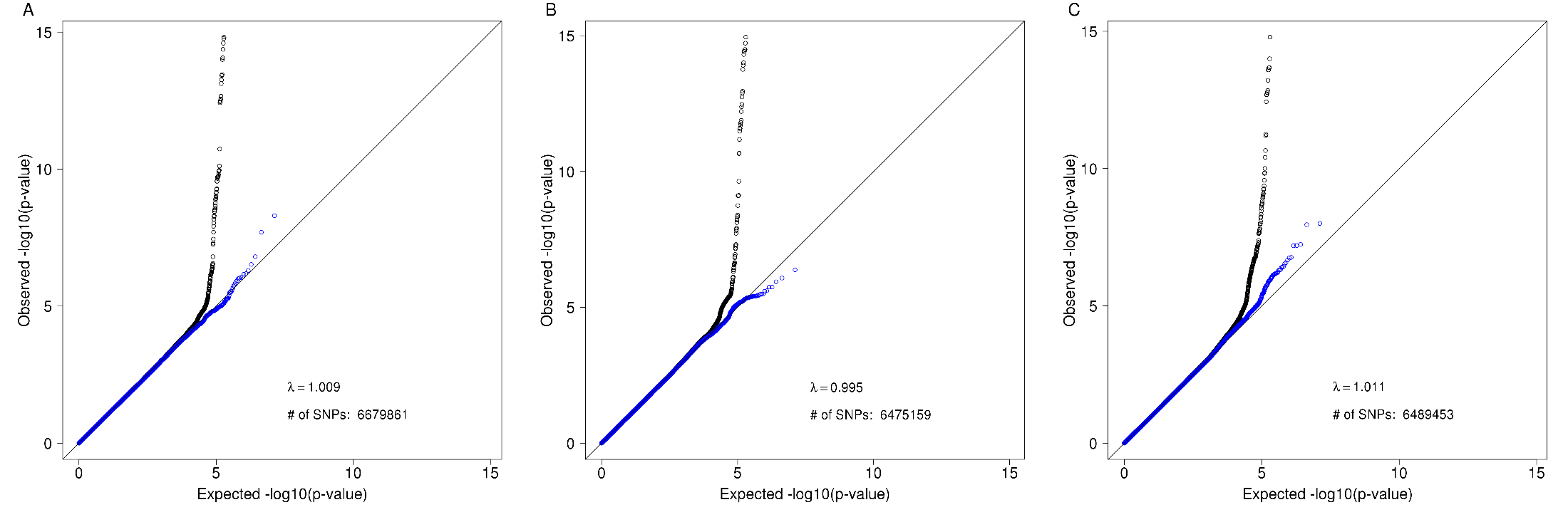


**Figure S2.** Manhattan plots showing genome-wide pleiotropy analyses of **(A)** NP and NFT, **(B)** NP and CAA, and **(C)** NFT and CAA using the O’Brien method. Red dot line represents the genome-wide significance threshold of P < 5.0x10^-8^. Loci achieving genome-wide significance are highlighted in red, and known Alzheimer’s disease genes which attained at least moderate significance level (P < 10^-4^) are highlighted in gold.


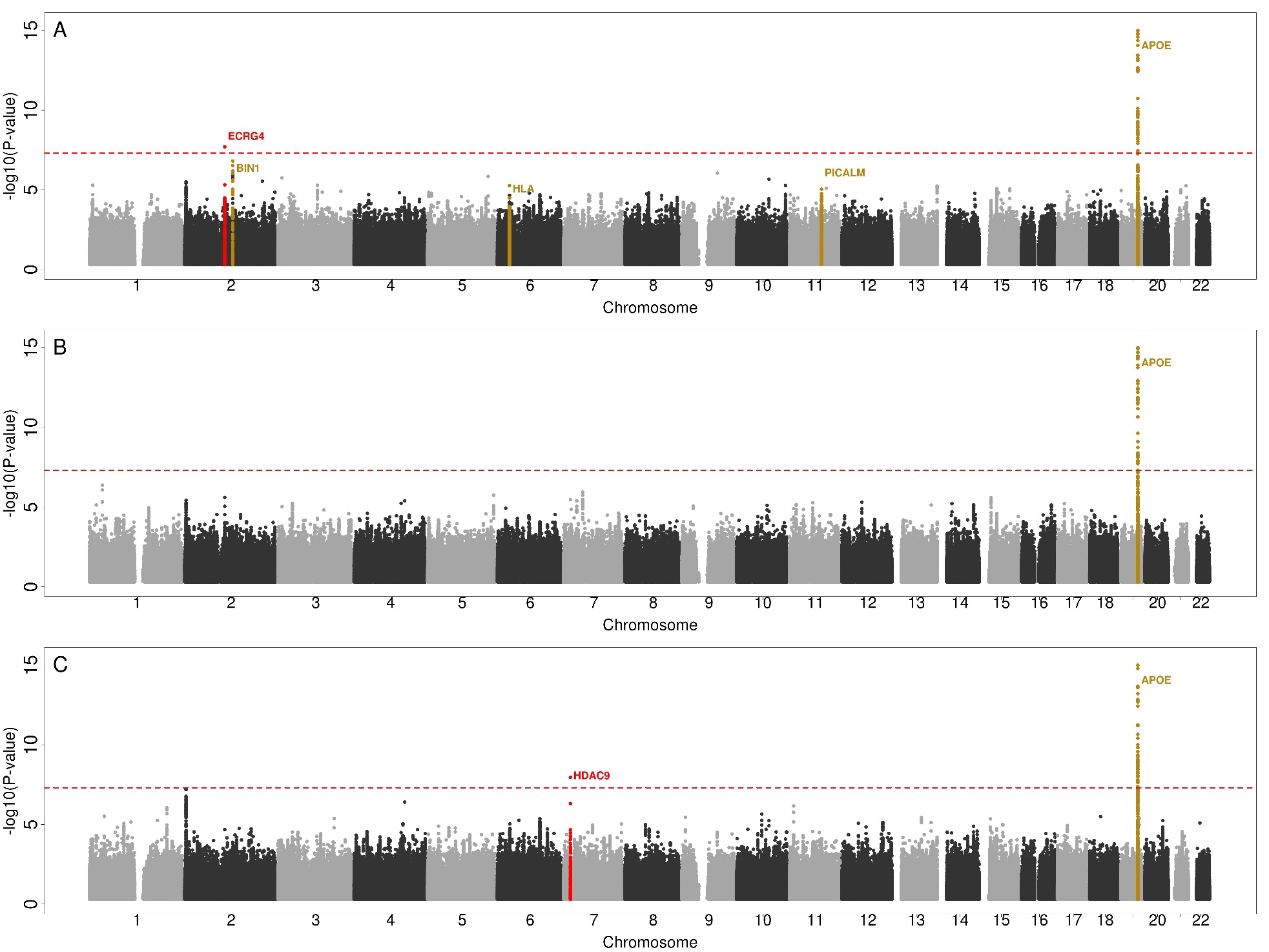


**Figure S3.** Regional association plots of genes including *TRAPPC12*, *TRAPPC12-AS1*, and *ADI1* on chromosome 2 from the joint model of NFT and CAA.

**
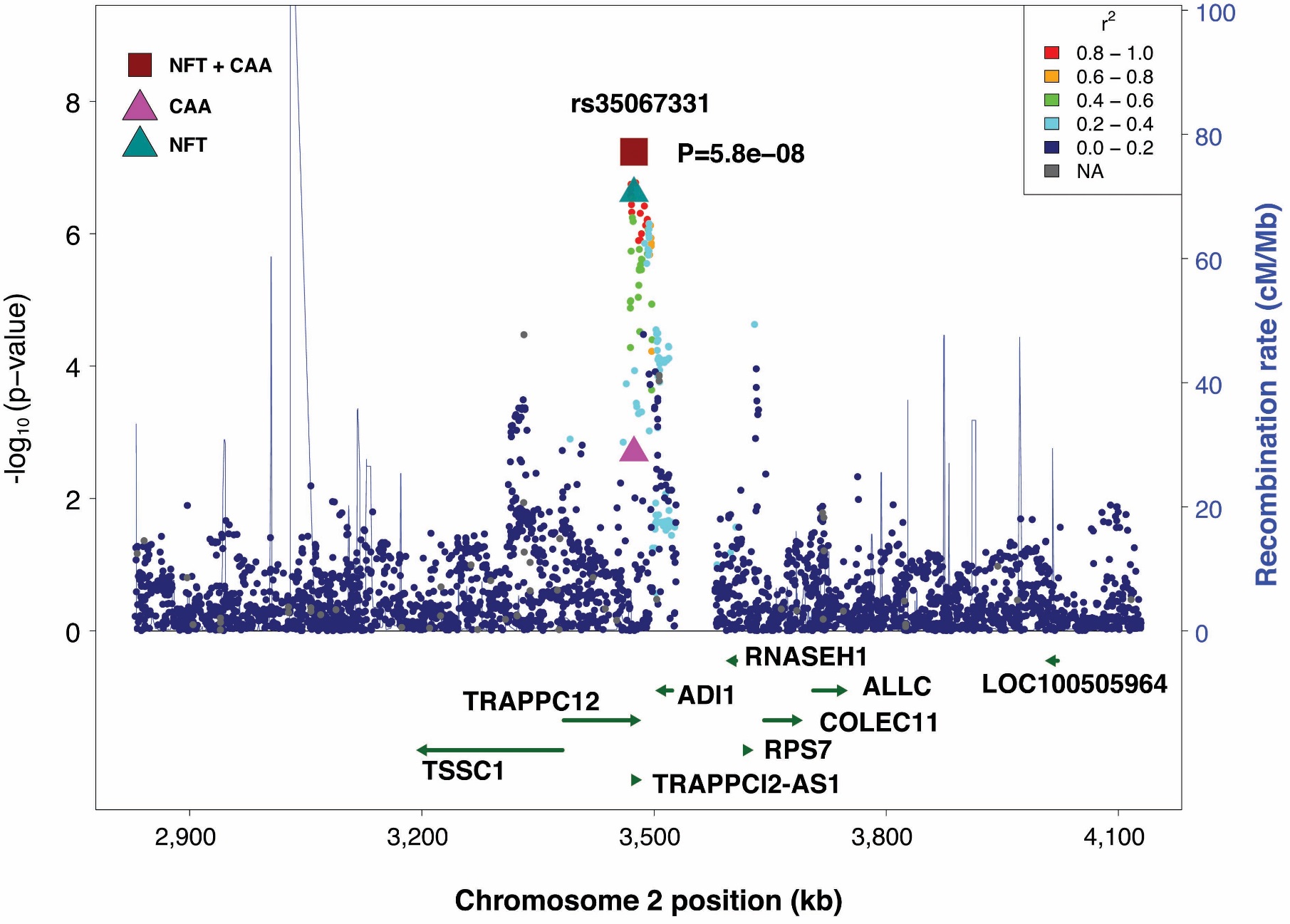
**

**Figure S4.** Genome-wide trivariate pleiotropy analysis of NP, NFT, and CAA. **(A)** Quantile-quantile plot. **(B)** Manhattan plot.

**A**

**
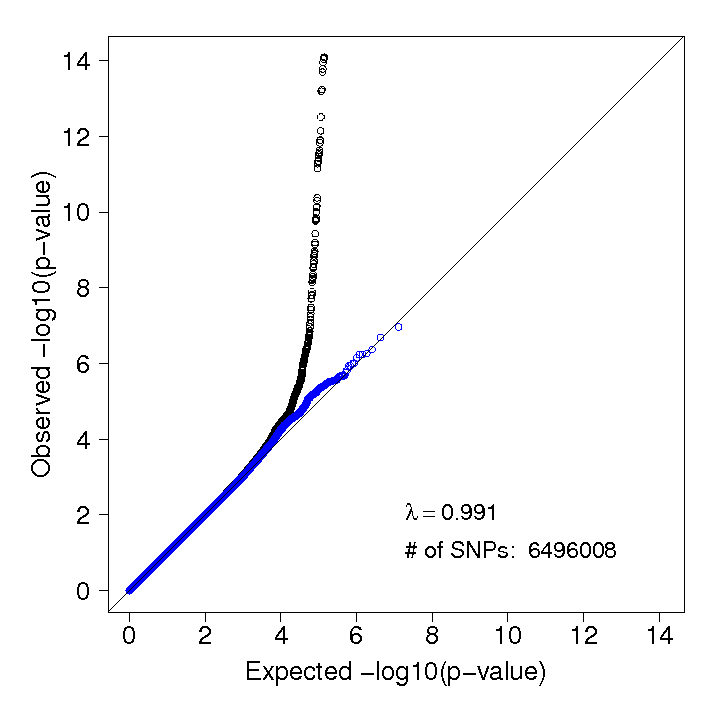
**

**B
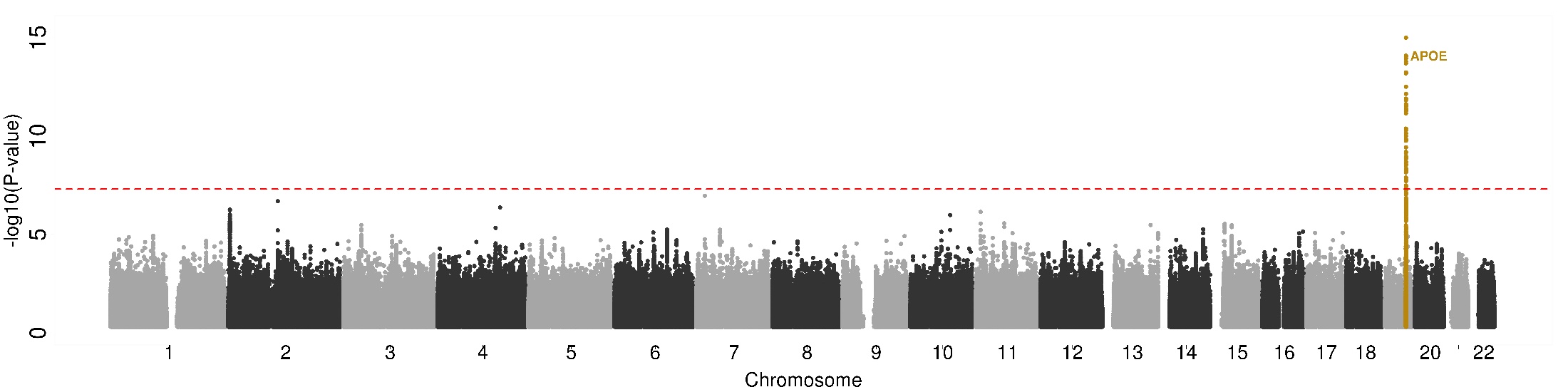
**
